# Generation and characterization of an anti ITGB4 monoclonal antibody that blocks Zika virus entry

**DOI:** 10.3389/fimmu.2026.1796913

**Published:** 2026-07-23

**Authors:** Xiaodong Han, Wenhui Li, Shuqian Yang, Yancun Gong, Jie Shen, Lei Song, Fang Wan, Guihua Wang, Haolong Cong

**Affiliations:** 1 College of Life Sciences, Inner Mongolia Agriculture University, Hohhot, Inner Mongolia, China; 2 Inner Mongolia Animal Disease Prevention and Control Center, Hohhot, Inner Mongolia, China; 3 Institute of Microbiology, Chinese Academy of Sciences, Beijing, China; 4 Chinese Academy of Quality and Inspection & Testing, Beijing, China

**Keywords:** ITGB4, monoclonal antibody, receptor blocking, viral entry, Zika virus

## Abstract

**Introduction:**

Zika virus (ZIKV) causes congenital disease and neurological complications, yet no approved vaccines or antivirals are available. Integrin β4 (ITGB4) has been identified as an entry receptor for ZIKV.

**Methods:**

Recombinant human ITGB4 ectodomain was expressed using a baculovirus-insect cell system and used to immunize BALB/c mice. Hybridoma screening identified monoclonal antibody 7C3. Binding affinity to ITGB4 was determined by biolayer interferometry. Epitope relationships between 7C3 and the previously reported anti-ITGB4 antibody 13H10 were assessed by competitive binding assay. Antiviral activity was evaluated in four cell lines by RT-qPCR quantification of cell-associated ZIKV RNA after antibody pretreatment and viral challenge.

**Results:**

7C3 bound ITGB4 with picomolar affinity (K_D_=30.9 pM) and high specificity. Competitive binding showed that 7C3 and 13H10 (K_D_=7.98 pM) recognize non-overlapping epitopes on ITGB4. Pretreatment with 7C3 significantly reduced cell-associated ZIKV RNA in all four cell lines compared with PBS and isotype controls.

**Discussion:**

A second, independently generated anti-ITGB4 antibody with picomolar affinity can robustly block ZIKV entry across multiple cell types, including SY5Y neuroblastoma cells relevant to congenital neuropathology. The non-overlapping epitopes of 7C3 and 13H10 suggest potential for combination strategies to enhance ITGB4 blockade. High germline identity of the 7C3 variable regions (V_H_=94.46%, V_L_=95.82%) supports future humanization. These findings establish 7C3 as a host-directed antiviral candidate and provide a basis for ITGB4-targeting approaches against congenital ZIKV disease.

## 1 Introduction

Zika virus (ZIKV) is a mosquito-borne flavivirus that causes congenital disease and neurological complications. It poses an ongoing global health threat (1). Infection during pregnancy can lead to severe fetal outcomes. These include microcephaly, intrauterine growth restriction, and fetal loss (2). ZIKV also causes neurological sequelae in adults, such as Guillain-Barré syndrome (3). Despite progress in understanding ZIKV pathogenesis, no widely approved vaccines or specific antiviral drugs exist. This highlights the urgent need for new therapeutic strategies (4).

ZIKV entry into host cells is a multistep process. It involves attachment factors on the cell surface and receptors that promote internalization and endosomal trafficking (5). Multiple host molecules contribute to ZIKV entry. Lectins and adhesion molecules such as DC-SIGN and Hsp70 facilitate viral attachment (6, 7). High-affinity entry receptors include integrin ITGB4, GRP78, and NCAM1 (8–10). Internalization-associated receptors such as integrin αvβ5 and sialic acid-containing glycans also participate (11, 12). Entry cofactors linked to endocytosis, including AXL and TIM-1, further contribute to the entry process (13). This diversity of entry pathways suggests a critical opportunity: host-directed interventions that block key receptor-virus interactions could complement direct-acting antivirals.

Among ZIKV entry receptors, ITGB4 has emerged as a particularly promising therapeutic target. Recent studies demonstrate that ITGB4 mediates viral attachment with high affinity and is functionally essential for infection across multiple cell types (14, 15). An anti-ITGB4 antibody (13H10, K_D_ = 7.98 pM) has been shown to bind ITGB4, block viral attachment, and reduce replication in permissive cells (10). It also protects pregnant mice and their fetuses from ZIKV infection. Antibodies or decoy proteins targeting other receptors, including NCAM1 and GRP78, suppress ZIKV entry in cellular models. TIM-1 blocking antibodies attenuate apoptotic mimicry-associated infection (8, 9). Antibodies targeting AXL or DC-SIGN limit infection in susceptible models and reduce dendritic cell-mediated dissemination (13). These findings establish proof of concept for receptor-blocking approaches. However, the therapeutic potential of ITGB4-targeting antibodies remains underexplored. Critical questions include whether additional high-affinity ITGB4 antibodies can be generated, whether they recognize conserved or distinct epitopes compared to 13H10, and whether picomolar-affinity binding translates to superior blocking activity across diverse cell types relevant to congenital disease.

Monoclonal antibodies (mAbs) offer a promising antiviral approach. They have high specificity and favorable safety profiles (16). Receptor-targeting antibodies may avoid antibody-dependent enhancement (ADE) concerns associated with some flavivirus-directed approaches (17). Unlike direct-acting antivirals that target viral proteins and may select for escape mutants, host-directed antibodies target conserved host factors (18). This reduces the likelihood of resistance development. For congenital ZIKV infection, where maternal antibodies can cross the placental barrier, receptor-blocking strategies represent a mechanistically distinct approach to prevent fetal transmission and neuropathology.

We hypothesized that immunization with highly pure, properly folded ITGB4 ectodomain would generate monoclonal antibodies with exceptional affinity capable of blocking ZIKV entry across multiple permissive cell types. To test this hypothesis, we expressed and purified the recombinant ITGB4 ectodomain using a baculovirus-insect cell system, which ensures high-yield production and native glycosylation patterns. Immunization of BALB/c mice with this antigen yielded monoclonal antibody 7C3. We characterized 7C3 by determining its isotype, sequencing variable regions, and annotating complementarity-determining regions (CDRs) and germline relationships using IMGT analysis. We quantified 7C3-ITGB4 binding kinetics by biolayer interferometry and evaluated ZIKV-blocking efficacy in four permissive cell lines, including neural cells directly relevant to congenital pathology. This work establishes the functional properties of 7C3 and provides a foundation for ITGB4-targeted host-directed interventions against congenital ZIKV disease.

## 2 Materials and methods

### 2.1 Ethics statement

All animals used in the experiments were handled according to the guidelines of the Institutional Animal Care and Use Committee of Inner Mongolia Agricultural University (protocol number: NND2021106). The Balb/c mice (6–8 weeks old) were purchased from Beijing Vital River Laboratory Animal Technology Co., Ltd. Mice were housed under specific pathogen-free conditions with a 12-hour light/dark cycle, controlled temperature (22 ± 2 °C), and ad libitum access to food and water.

### 2.2 Cells, virus, and antibody

African green monkey kidney epithelial cells (Vero), human hepatocellular carcinoma cells (Huh-7, ATCC HB-8065), human neuroblastoma cells (SY5Y, ATCC CRL-2266), baby hamster kidney fibroblasts (BHK-21), and human lung adenocarcinoma cells (A549) were maintained in Dulbecco’s modified Eagle’s medium (DMEM; Gibco). DMEM was supplemented with 10% heat-inactivated fetal bovine serum (FBS; Gibco), 100 U/mL penicillin, and 100 μg/mL streptomycin. Cells were cultured at 37 °C in a humidified atmosphere with 5% CO_2_.

ZIKV strain MR766 (GenBank: MK105975.1) was provided by Dr. Shihua Li (Institute of Microbiology, Chinese Academy of Sciences). Virus stocks were propagated in Vero cells and titrated by the Reed-Muench method. Plasmid pcDNA3.1/*Myc-His-beta4* that contained full length *ITGB4* expression were obtained from Addgene(#16039). Commercial isotype control mAb (P3.6.2.8.1) was purchased from Thermo Fisher Scientific. The 13H10 antibody was obtained as previously described (10). Purified 13H10 was labeled with Cy5 using a Cy5 Conjugation Kit (Fast)-Lightning-Link (Abcam, #ab188288) according to the manufacturer’s instructions.

### 2.3 Construction of expression vectors and Bacmid

The extracellular segment (Asp28-Asn740) of the human *ITGB4* gene (GenBank: NM_000213.5) was amplified by PCR using the plasmid pcDNA3.1/*Myc-His-beta4* as template. Following digestion with the restriction endonucleases *Not*I and *Sal*I, the PCR-amplified fragment was ligated into the equivalently digested pFastBac1 expression vector (Novagen, USA), which harbors an N-terminal GP67 signal sequence and a C-terminal polyhistidine (6×His) tag.

The recombinant pFastBac1-*ITGB4* plasmid was verified by DNA sequencing (Sangon Biotech, China). It was transformed into DH10Bac competent cells (Invitrogen) according to the Bac-to-Bac^®^ Baculovirus Expression System protocol. Transformants were plated on LB agar. The agar contained kanamycin (50 μg/mL), gentamicin (7 μg/mL), tetracycline (10 μg/mL), X-gal (100 μg/mL), and IPTG (40 μg/mL) for blue-white screening. White colonies indicated successful transposition. They were selected after 48 h incubation at 37 °C. Positive clones were verified by PCR using M13 forward and M13 reverse primers. Bacmid DNA was isolated using a NucleoBond^®^ Xtra Maxi EF Kit (Macherey-Nagel) and stored at -80 °C.

### 2.4 Production of recombinant baculoviruses

Sf9 cells (8-9 × 10^5^ cells/well) were transfected with recombinant bacmid DNA (2-4 μg) and Lipofectamine 3000 (6-8 μL) at 27 °C for 4–6 h, then cultured in antibiotic supplemented medium. At 72 h post-transfection, supernatants showing cytopathic effects were collected as P1 viral stock. P1 virus was serially diluted (10^-5^, 10^-6^, 10^-7^) and used to infect Sf9 cells. After viral adsorption, cells were overlaid with 4% agarose–medium and incubated at 27 °C for 7–10 days for plaque counting and titer determination. For amplification, P1 virus sequentially infected Sf9 cells at MOI 0.001–0.01 to generate P2 and P3 stocks (72 h each), and P3 virus was further amplified in suspension culture (2 × 10^-6^ cells/mL) to produce P4 viral stock.

### 2.5 Protein expression and purification.

Hi5 cells (2.0-2.5 × 10_-6_ cells/mL) were infected with P4 recombinant baculovirus at an MOI of 2.5 and cultured at 27 °C for 48 h. Culture supernatant was collected by centrifugation (7000 × g, 30 min, 4 °C) and filtered through a 0.22 μm membrane. The clarified supernatant was loaded onto a HisTrap HP column (5 mL) and eluted with a linear gradient of imidazole (0–1 M) in buffer (20 mM Tris-HCl, 200 mM NaCl, pH 8.0). ITGB4-containing fractions were pooled and concentrated to 2ml. Final purification was achieved by size-exclusion chromatography (SEC) using a Superdex 200 PG column (Cytiva, USA) equilibrated with buffer containing 25 mM Tris-HCl (pH8.0), and 200 mM NaCl. ITGB4 was pooled and concentrated using an Amicon Ultra-15 filter unit (Millipore, USA). Protein purity was assessed by 10% Coomassie-stained SDS-PAGE, and protein concentration was determined using the Bradford assay with bovine serum albumin (BSA) as the standard.

### 2.6 Generation of hybridomas, collection of mouse ascites, and subclass determination of antibody

Balb/c mice (6–8 weeks old) were immunized with 50 μg purified ITGB4 protein in complete Freund’s adjuvant by subcutaneous injection, followed by a second immunization on day 21 with 50 μg ITGB4 in incomplete Freund’s adjuvant via the same route. On day 42, a third immunization was administered by intraperitoneal injection of 50 μg ITGB4 without adjuvant, and a booster immunization with 500 μg ITGB4 was given on day 56. Three days after the booster, mice were euthanized by CO_2_ inhalation at a displacement rate of 30-70% of the chamber volume per minute, followed by cervical dislocation to confirm death, and spleens were aseptically collected. Splenocytes were fused with SP2/0 myeloma cells using standard hybridoma technology, and ten days later, positive clones secreting anti-ITGB4 antibodies were subcloned to monoclonality. Antibody titers in serum and cell culture supernatant were determined by indirect ELISA, and the antibody subclass was identified using an isotype-specific assay kit.

For ascites production, an eight-week-old BALB/c mouse was pre-conditioned by intraperitoneal injection of 0.5 mL tetramethylpentadecane 7 days before hybridoma transfer. Hybridoma cells (5×10^6^ in 0.5 mL PBS) were then injected intraperitoneally, and ascites fluid was collected by peritoneal lavage 10 days later following euthanasia as described above.

### 2.7 Cloning and sequencing of IgG VH and VL genes

Total RNA from hybridoma clone 7C3 (producing antibodies against ITGB4) was extracted, and cDNA was synthesized using HiFiScript gDNA Removal cDNA Synthesis Kit (Cwbio). The immunoglobulin heavy chain variable region (VH) was amplified by PCR using forward primer 5’-GGG GAT ATC CAC CAT GRA CTT CGG GYT GAG CTK GGT TTT-3’ and reverse primer 5’-GAC HGA TGG GGS TGT YGT GCT AGC TGM RGA GAC DGT GA-3’. The light chain variable region (VL) was amplified using forward primer 5’-GGG GAT ATC CAC CAT GGA GAC AGA CAC ACT CCT GCT AT-3’ and reverse primer 5’-GGA TAC AGT TGG TGC AGT CGA CTT ACG TTT KAT TTC CAR CTT-3’. PCR products for both VH and VL regions were cloned and sequenced, and the sequences were submitted to the International ImMunoGeneTics information system (IMGT)/V-QUEST website to identify the V and J segments of the immunoglobulin genes.

### 2.8 BioLayer interferometry

The affinity between 7C3 mAb and ITGB4 was measured by BLI on an OctetRed 96 (ForteBio). The ITGB4 protein was randomly biotinylated on surface-exposed lysine side chains with succinimidyl-6-(biotinamido) hexanoate, and then purified using a ZebaSpin desalting column (Fisher, #89882) and diluted to 50 µg/ml. The sensors (super streptavidin) were pre-wet in dialysis buffer for 15 min before use and then loaded with biotinylated ITGB4 proteins for 15 min. 7C3 mAb was serially diluted (78.2, 156.3, 312.5, 625, 1250, and 2500 pM) in a 96-well plate. The measurements were carried out automatically at room temperature. Experiments were performed in three biological replicates with curve fitting using a 2:1 (Heterogeneous Ligand) model. Sensorgram quality was assessed visually and the replicates with residuals exceeding 10% of the maximum response were excluded from kinetic analysis.

### 2.9 Competitive binding assay

Recombinant ITGB4 ectodomain (2 µg/mL in PBS) was coated onto 96-well MaxiSorp plates overnight at 4 °C. The plates were washed with PBS containing 0.05% Tween-20 (PBS-T) and blocked with 3% BSA in PBS-T for 1 h at room temperature (RT). For the competitive binding assay, Cy5-labeled 13H10 was prepared as a serial dilution and added to the ITGB4-coated wells either alone or together with unlabeled 7C3. Unlabeled 7C3 was mixed with Cy5-labeled 13H10 immediately before addition to the wells and incubated for 1 h at RT to allow binding. After five washes with PBS-T to remove unbound antibodies, Cy5 fluorescence was measured using a fluorescence microplate reader at excitation/emission wavelengths of 650/670 nm. Relative fluorescence intensity (RFI) was calculated and expressed as a percentage of the signal obtained with Cy5-labeled 13H10 alone, which served as the control condition.

### 2.10 ZIKV infection inhibition assay

A549, Huh-7, BHK-21, and SY5Y cells (2 × 10^5^ cells/well) were pre-incubated with 7C3 or an isotype-control antibody (10 μg/mL) for 1 h at 4 °C to allow antibody binding to the cell surface. Cells were then exposed to ZIKV MR766 at a multiplicity of infection (MOI) of 1 at 4 °C for 1 h to permit viral binding. After incubation, cells were washed three times with ice-cold PBS to remove unbound ZIKV. Fresh culture medium containing the corresponding antibody (10 μg/mL) was then added, and cells were incubated at 37 °C in 5% CO_2_ for 48 h to permit viral replication. Culture supernatant (100 μL) was collected, and total RNA was extracted using TRIzol or equivalent extraction reagent. Viral RNA copy numbers were quantified by real-time RT-qPCR using ZIKV-specific primers targeting the viral E gene.

## 3 Results

### 3.1 Expression and purification of the ITGB4 ectodomain

**Figure 1:**
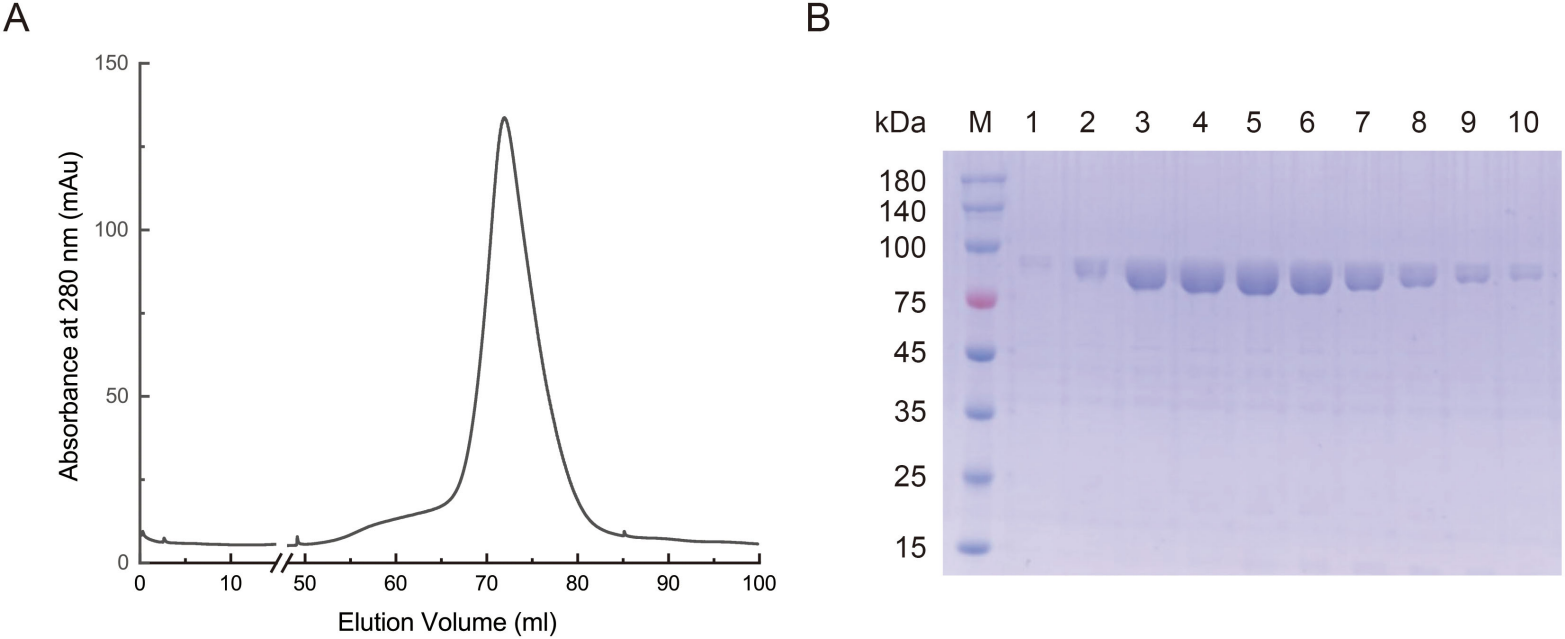
Expression and purification of recombinant ITGB4 ectodomain. **(A)** SEC profile of the ITGB4 ectodomain on a Superdex 200 PG column. **(B)** Coomassie-stained SDS-PAGE of SEC fractions. Lanes 1–10 show fractions from the main SEC peak, with a single band at 82 kDa and >95% purity.

Following Ni-affinity chromatography and size-exclusion chromatography, the ITGB4 ectodomain eluted as a single sharp peak at an elution volume of 72 mL (**Figure 1A**). The elution volume corresponds to an apparent molecular weight of approximately 160 kDa based on calibration with gel filtration standards. This suggests that ITGB4 exists as a homodimer in solution under these buffer conditions. SDS-PAGE analysis showed a single major band at the expected monomer size of approximately 82 kDa, with purity exceeding 95% as assessed by densitometry (**Figure 1B**). Peak fractions were pooled and concentrated to 10 mg/mL as determined by the Bradford assay.

### 3.2 Hybridoma immunization, screening, and monoclonal antibody production

**Figure 2:**
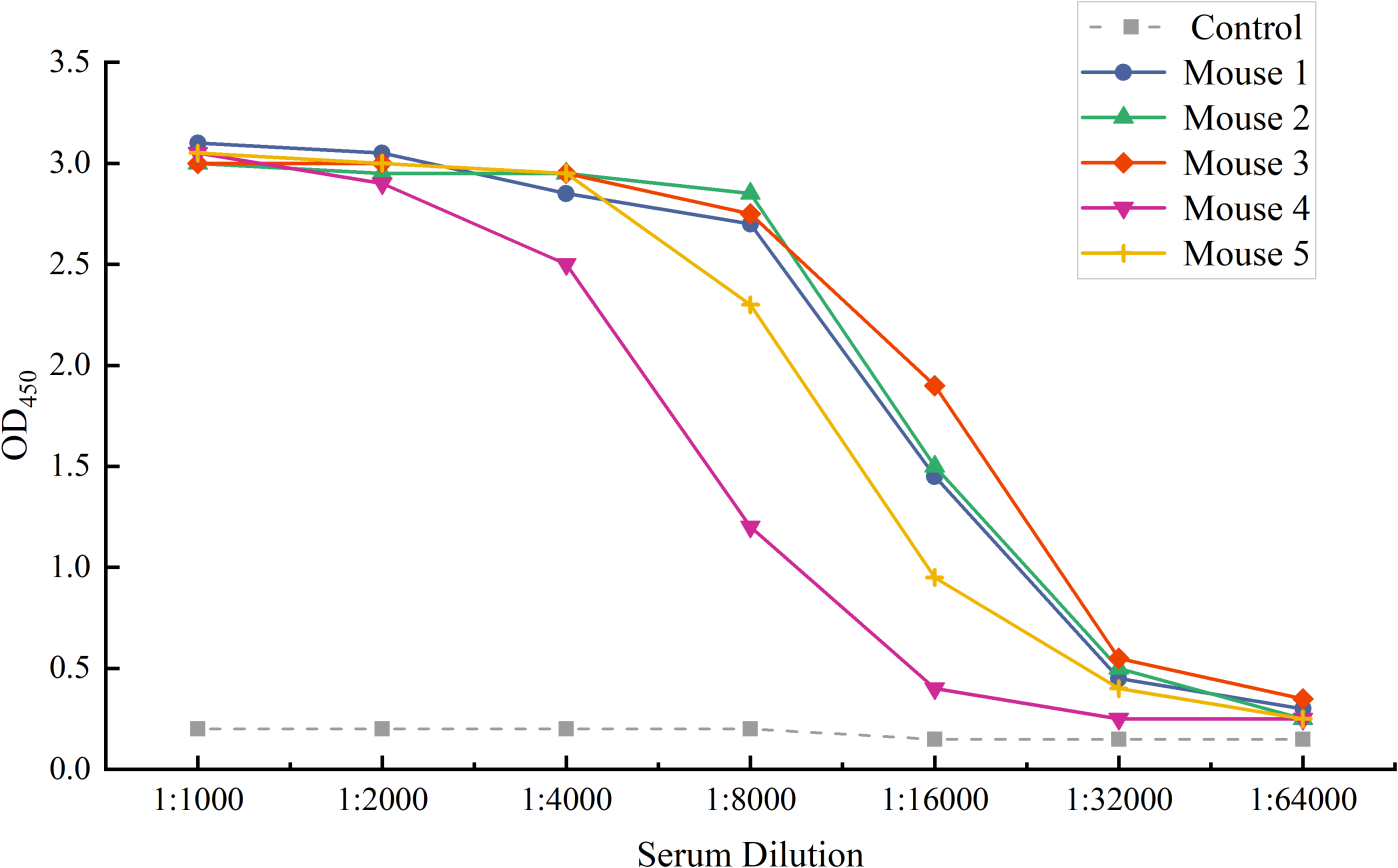
Serum antibody titers in ITGB4-immunized mice detected by ELISA. BALB/c mice (n = 5) were immunized with recombinant ITGB4 ectodomain protein. Serum samples collected after the final boost were serially diluted (1:1, 000 to 1:64, 000) and tested by indirect ELISA against ITGB4-coated plates. Absorbance was measured at 450 nm (OD_450_). The dotted line indicates the positivity cutoff (2× background OD of non-immunized control serum). Each colored line represents an individual mouse.

Immunization of BALB/c mice with purified ITGB4 ectodomain elicited a robust humoral immune response. Following four immunization cycles over 56 days, mouse sera were analyzed by indirect ELISA using plates coated with 1 μg/mL ITGB4. All five immunized mice achieved endpoint titers of 1:64, 000 or higher. Mouse #3 displayed the highest anti-ITGB4 antibody titers across all dilutions tested (**Figure 2**). Based on this superior response, Mouse #3 was selected for an additional booster immunization and subsequent splenocyte harvest.

Three days after the final booster, splenocytes from Mouse #3 were fused with SP2/0 myeloma cells at a 5:1 ratio using standard hybridoma technology. Fused cells were distributed into 96-well plates and cultured in HAT selection medium. Screening of fusion-derived cell lines at 14 days post-fusion by indirect ELISA identified 78 wells containing hybridomas that secreted ITGB4-reactive IgG antibodies. Positive hybridoma clones were subjected to two rounds of limiting dilution subcloning to ensure monoclonality. This yielded 46 stable monoclonal producing cell lines.

Among the 46 stable clones, clone 7C3 demonstrated the highest anti-ITGB4 antibody titer in both culture supernatant and mouse ascites, as determined by indirect ELISA. ELISA screening data for all 46 stable clones are provided in **Supplementary Table 1**, confirming that 7C3 exhibited the highest OD_450_ value (3.45 at 1:1000 dilution) among all clones tested (**Supplementary Table 1**). Isotype-specific immunoassay using a Rapid Mouse Antibody Isotyping Kit confirmed that 7C3 produces an IgG isotype antibody with kappa light chains. The IgG isotype is well-suited for receptor-blocking therapeutic applications owing to its favorable pharmacokinetic properties and efficient Fc-mediated effector functions.

### 3.3 Sequence, specificity, and epitope characterization of 7C3

**Figure 3:**
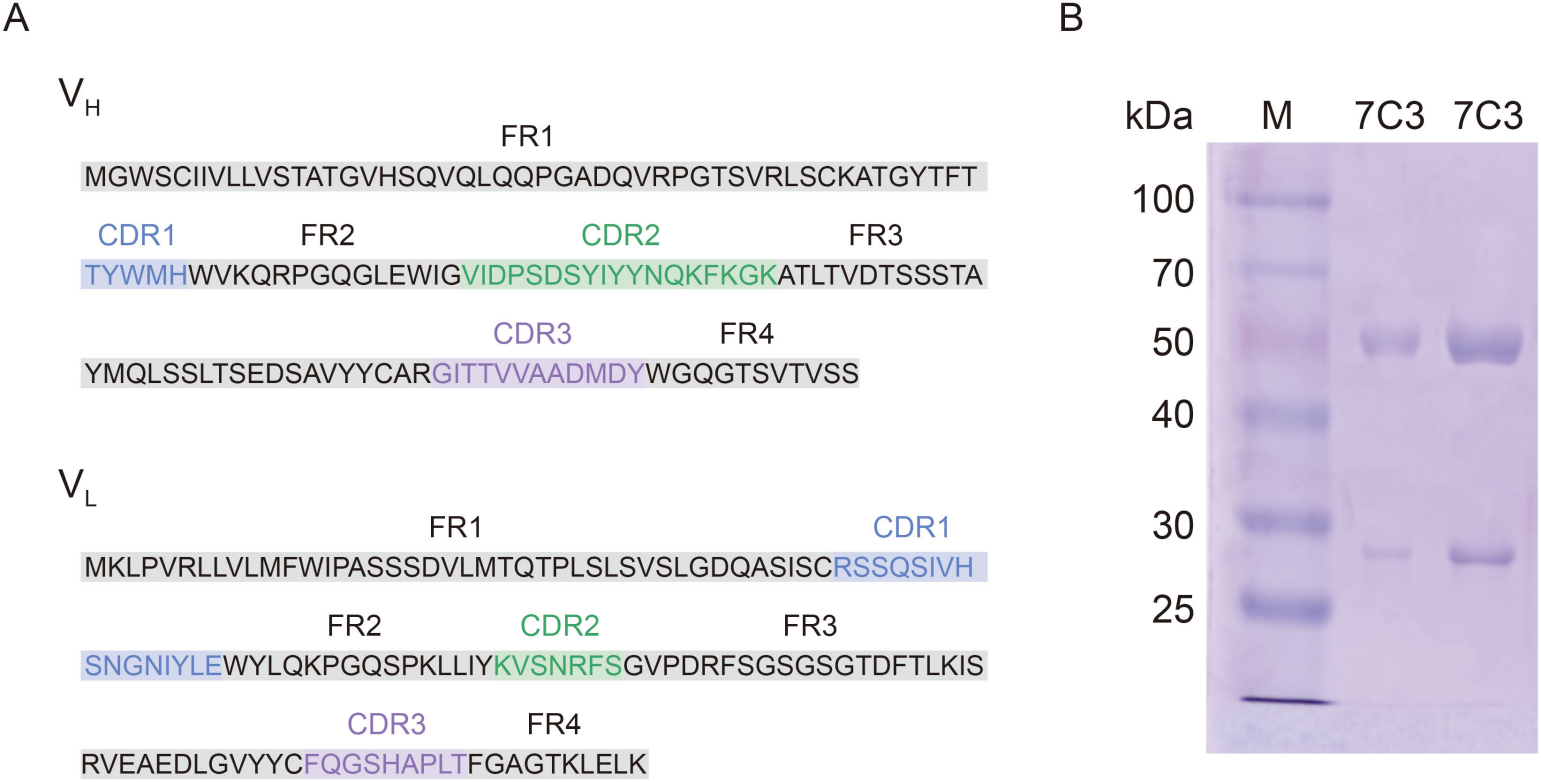
Sequence and purification of 7C3. **(A)** Variable region sequences of 7C3 V_H_ and V_L_. The FR1, FR2, FR3, and FR4 segments were colored black. CDR1 was colored red. Framework regions (FR1–FR4) are shown in black. Complementarity-determining regions (CDRs) are highlighted. **(B)** The purified 7C3 antibody was detected by 12% SDS-PAGE. Lane M, molecular weight markers (kDa).

The immunoglobulin variable regions of 7C3 were successfully amplified from hybridoma cDNA, cloned, and sequenced. After sequencing of 7C3, we performed a BLAST analysis against mouse germline genes using the IMGT/V-QUEST tool. The results showed that the variable regions in V_H_ and V_L_ of 7C3 shared 94.46% and 95.82% identity to the original sequences of V gene, suggesting that V_H_ and V_L_ of 7C3 underwent limited somatic hypermutation during affinity maturation, consistent with the typical 5-10% sequence divergence from germline observed in antigen-experienced B cells (19). The identified V gene segments of V_H_ and V_L_, and CDR sequences of the heavy and light chain are shown in **Figure 3A**.

Large amounts of 7C3 antibody were obtained from mouse ascites and purified by saturated ammonium sulfate precipitation followed by Protein-G affinity chromatography, yielding highly pure monoclonal IgG. The purity of 7C3 was confirmed by SDS−PAGE, which showed a heavy chain band at 55 kDa and a light chain band at 27 kDa, consistent with a standard IgG structure (**Figure 3B**).

### 3.4 7C3 binds ITGB4 with high affinity

**Figure 4:**
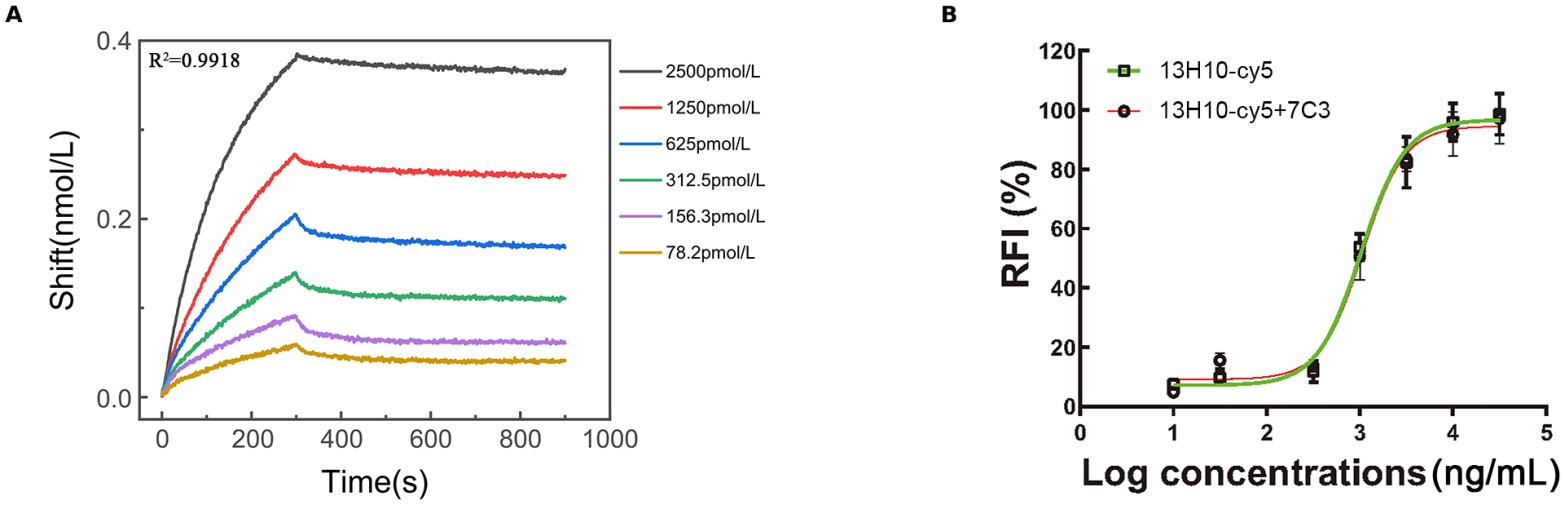
Octet BLI analysis of 7C3 binding to immobilized ITGB4. **(A)** Sensorgrams were obtained by flowing serial dilutions of 7C3 over biotinylated ITGB4 captured on streptavidin biosensors. **(B)** Competitive binding of 7C3 and 13H10 to ITGB4. ITGB4−coated plates were incubated with serial dilutions of Cy5−labeled 13H10 in the absence or presence of unlabeled 7C3. The relative fluorescence signal (RFI, %) was measured at Ex/Em 650/670 nm, and concentration–response curves were fitted using a four−parameter logistic (4PL) model.

Global fitting with a 2:1 heterogeneous ligand model yielded a dissociation constant (K_D_) of 30.9 pM, indicating high-affinity binding between 7C3 and ITGB4. Curve fitting quality was excellent; residuals were uniformly <5% of the maximum response across all analyte concentrations (**Figure 4A**). Curve fitting quality was excellent, with minimal deviation between experimental data and the fitted model. Under identical experimental conditions, the isotype control antibody showed no detectable binding to ITGB4 (response <0.05 nm), confirming that the interaction is specific to 7C3.

Given that 7C3 and 13H10 bind ITGB4 with equilibrium dissociation constants (K_D_) of 30.9 pM and 7.98 pM respectively (as previously reported), we next investigated whether 7C3 competes with 13H10 for ITGB4 binding. We examined the binding of Cy5-labeled 13H10 to immobilized ITGB4 in the absence or presence of unlabeled 7C3. Cy5-labeled 13H10 alone produced a typical sigmoidal concentration-response curve, with RFI increasing as the antibody concentration increased. In the presence of unlabeled 7C3, the binding curve of Cy5-13H10 closely overlapped with that obtained for Cy5-13H10 alone across the tested concentration range. No obvious rightward shift in the curve or decrease in maximal RFI was observed when 7C3 was included (**Figure 4B**). These findings indicate that 7C3 does not measurably compete with 13H10 for ITGB4 binding under these assay conditions, suggesting that the two antibodies likely recognize distinct or non-overlapping epitopes on ITGB4.

### 3.5 7C3 blocks ZIKV entering cells

**Figure 5:**
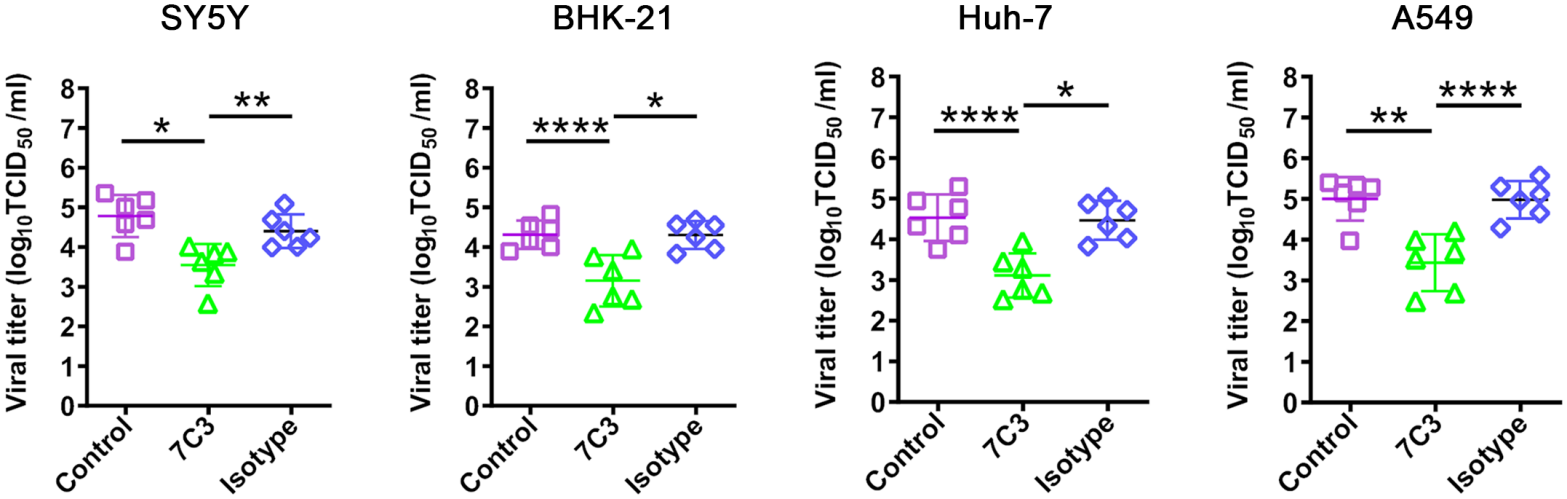
RT-PCR analysis showing the binding of ZIKV to SY5Y, BHK-21, Huh-7, and A549 cells. Four cell lines (SY5Y, BHK-21, Huh-7, A549) were pre-treated with PBS, isotype control, or 7C3 (10 µg/mL) prior to ZIKV MR766 infection (MOI = 1). Viral RNA was quantified by RT-qPCR targeting the E gene; error bars represent ± SD, n = 5. Significance was determined by one-way ANOVA with Tukey’s *post hoc* test, ****p < 0.0001.

To determine whether 7C3 functionally blocks ZIKV entry, we conducted infection inhibition assays in SY5Y, BHK-21, Huh-7, and A549 cell lines. The results showed that 7C3 pretreatment significantly reduced cell-associated ZIKV RNA across all four cell types compared to PBS and isotype control groups, as evidenced by decreased viral RNA copies. This consistent reduction in cell-associated ZIKV genomes demonstrates that 7C3 engagement of ITGB4 significantly reduces ZIKV infection, consistent with inhibition of an early ITGB4-mediated entry step (**Figure 5**).

## 4 Discussion

This study describes the generation and characterization of 7C3, a monoclonal antibody targeting the ITGB4 ectodomain, and demonstrates that it efficiently blocks ZIKV entry in multiple permissive cell lines. 7C3 binds ITGB4 with picomolar affinity (K_D_ = 30.9 pM) by biolayer interferometry and shows no detectable binding of an isotype control, confirming high specificity. In infection inhibition assays, 7C3 pretreatment significantly reduced cell-associated ZIKV RNA in SY5Y, BHK-21, Huh-7, and A549 cells compared with PBS and isotype control groups, indicating that engagement of ITGB4 by 7C3 blocks an early step of viral entry. These findings are consistent with the established role of ITGB4 as a ZIKV entry receptor and extend this work by introducing 7C3 that effectively blocks ZIKV entry in diverse cellular contexts.

These data build on previous work identifying ITGB4 as a high-affinity entry receptor for ZIKV and showing that the anti−ITGB4 antibody 13H10 (K_D_ = 7.98 pM) protects cells and pregnant mice from infection (10). The present results show that a second, independently generated anti−ITGB4 antibody with picomolar affinity can reproduce robust blocking activity across four cell lines, including SY5Y neuroblastoma cells that are relevant to congenital neuropathology. The consistent antiviral effect of 7C3 in both neural and non−neural cells further supports ITGB4 as a therapeutically relevant receptor. The variable regions of 7C3 retain high identity to mouse germline sequences (94.46% for V_H_ and 95.82% for V_L_), suggesting that a largely germline−encoded CDR framework can mediate high−affinity ITGB4 recognition and may facilitate subsequent humanization (20, 21).

An important finding is that 7C3 and 13H10 do not compete for the same epitope on ITGB4. In a competitive binding assay, Cy5−labeled 13H10 showed an unchanged concentration-response curve and maximal signal in the presence of unlabeled 7C3, indicating non−overlapping binding sites. This non−competition suggests that 7C3 and 13H10 could be combined to simultaneously engage distinct ITGB4 epitopes and potentially enhance blockade of viral attachment, as has been demonstrated for non-competing antibody cocktails against other viruses (22, 23). It also implies that the two antibodies may interact with different functional domains of ITGB4, which could differentially influence virus-receptor interactions or downstream signaling. Structural epitope mapping will be valuable for defining the basis of this relationship and rationally designing combination regimens.

Targeting a host receptor instead of a viral antigen offers conceptual advantages, including reduced selective pressure on viral proteins and a higher barrier to resistance (24, 25). This concept is consistent with broader experience from host−directed antiviral therapies, in which antibodies or inhibitors against host factors can limit escape driven by viral mutation. At the same time, ZIKV can use multiple entry factors such as integrin αvβ5, sialic acid–containing glycans, AXL, and TIM−1, and host−directed antibodies must be evaluated with respect to receptor biology, tissue context, and Fc−mediated effects. ZIKV preferentially infects human neural progenitor cells and placental trophoblasts (26, 27), and future studies should prioritize these primary cell types to better model congenital pathology. Overall, 7C3 emerges as a high-affinity, ITGB4-specific antibody that blocks ZIKV entry across several cell types and recognizes an epitope distinct from that of 13H10, supporting its further development as a host−directed antiviral candidate and as a component of ITGB4−targeting combination strategies against congenital ZIKV infection.

## Data Availability

The original contributions presented in the study are included in the article/**Supplementary Material**. Further inquiries can be directed to the corresponding authors.
