# Supplementary material for: Generation and characterization of an anti ITGB4 monoclonal antibody that blocks Zika virus entry
Source: Front Immunol. 2026 Jul 23;17:1796913. doi: 10.3389/fimmu.2026.1796913 (PMC13442645; doi:10.3389/fimmu.2026.1796913)
Supplement: Supplementary file 1 [file Table1.docx]

**Supplementary Table S1.** ELISA screening of 46 hybridoma clones against recombinant ITGB4 ectodomain.

| **Clone ID** | **OD₄₅₀ (raw)** | **OD₄₅₀ (blank-corrected)** |
| --- | --- | --- |
| 1-A6 | 2.3544 | 2.3064 |
| 1-A9 | 1.9480 | 1.9000 |
| 1-B2 | 1.4673 | 1.4193 |
| 1-B8 | 3.2246 | 3.1766 |
| 1-B9 | 1.4927 | 1.4447 |
| 1-C3 | 1.6900 | 1.6420 |
| 1-C6 | 2.7318 | 2.6838 |
| 1-G3 | 1.9515 | 1.9035 |
| 1-G5 | 1.3772 | 1.3292 |
| 1-H3 | 3.2910 | 3.2430 |
| 1-H4 | 1.3038 | 1.2558 |
| 1-H5 | 2.9512 | 2.9032 |
| 1-H7 | 2.0296 | 1.9816 |
| 2-D1 | 1.6541 | 1.6061 |
| 3-D8 | 3.2474 | 3.1994 |
| 3-F10 | 2.9067 | 2.8587 |
| 3-G8 | 1.8846 | 1.8366 |
| 4-A7 | 1.9659 | 1.9179 |
| 4-A8 | 2.2397 | 2.1917 |
| 4-C3 | 1.3331 | 1.2851 |
| 4-D11 | 2.0044 | 1.9564 |
| 4-E2 | 2.3022 | 2.2542 |
| 4-H11 | 3.2755 | 3.2275 |
| 5-H4 | 2.8814 | 2.8334 |
| 5-H5 | 1.7705 | 1.7225 |
| 5-H9 | 1.5984 | 1.5504 |
| 6-C8 | 3.0214 | 2.9734 |
| 6-E10 | 3.3307 | 3.2827 |
| **7C3 *** | **3.5000** | **3.4520** |
| 7-E5 | 2.1835 | 2.1355 |
| 7-G2 | 2.1032 | 2.0552 |
| 8-C8 | 2.9966 | 2.9486 |
| 8-F10 | 2.1866 | 2.1386 |
| 9-C5 | 1.4990 | 1.4510 |
| 9-E2 | 3.0675 | 3.0195 |
| 9-G11 | 1.4250 | 1.3770 |
| 9-H3 | 2.9021 | 2.8541 |
| 10-A4 | 3.0256 | 2.9776 |
| 10-B11 | 2.1320 | 2.0840 |
| 10-D5 | 3.1882 | 3.1402 |
| 10-D6 | 1.8995 | 1.8515 |
| 10-D11 | 2.6743 | 2.6263 |
| 11-F7 | 2.5003 | 2.4523 |
| 11-G5 | 2.4926 | 2.4446 |
| 12-C8 | 2.7482 | 2.7002 |
| 12-E1 | 2.1375 | 2.0895 |

** Clone 7C3 (highest OD₄₅₀ among all 46 hybridoma clones) was selected for further characterization. Coating antigen: recombinant ITGB4 ectodomain (1 μg/mL in PBS, overnight at 4°C). Culture supernatant dilution: 1:100. Blank-corrected OD₄₅₀ = raw OD₄₅₀ − mean empty-well OD₄₅₀.*
